# Neurocognitive psychotherapy for adult attention deficit hyperactive disorder

**DOI:** 10.4103/0972-6748.62271

**Published:** 2009

**Authors:** Susmita Halder, Akash Kumar Mahato

**Affiliations:** Department of Psychiatry, Manipal College of Medical Science, Pokhra, Nepal; 1 Clinical Psychology, LGB, Regional Institute of Mental, Health, Tezpur, Assam, India

**Keywords:** Attention-deficit hyperactivity disorder, Neurocognitive psychotherapy, Psychological management

## Abstract

Previously thought as a childhood disorder, attention-deficit hyperactivity disorder (ADHD) is reported to be spreading at an increasing rate and affecting 4% to 5% of the adult population. It is characterized by persistent problems of inattention, hyperactivity and impulsivity. We present the case of an adult ADHD patient intervened with neurocognitive psychotherapy.

Attention-deficit hyperactivity disorder (ADHD) typically develops during early childhood and often persists through adolescence and into adulthood. Indeed, for some patients, symptoms of ADHD do not become problematic until the individual is faced with the complex challenges of adulthood. ADHD is characterized by persistent problems of inattention, hyperactivity and impulsivity. These core symptoms lead to a wide range of impairments in adulthood, including lower educational attainment, lower vocational achievement, difficulties in interpersonal relationships and high risk for a wide range of additional psychiatric comorbidities. Once thought to be exclusively a disorder of childhood, it is now estimated that 4% of adults have ADHD (Hesslinger *et al*., 2002; Kessler *et al*., 2006). In spite of patients being almost normal or showing average functions in other domains, symptoms of poor and ill-sustained attention, disorganization and forgetfulness negatively affect the daily functioning of adults at the workplace and at home in numerous ways. Similarly, problems with organization often result in repeated loss or misplacement of items, inefficiency of effort and a disorderly workplace or home environment, also producing stress for oneself and one’s spouse or partner. It has generally been found that adult patients of ADHD resort to consultation only when it starts hampering their functioning level, with complaints focusing on poor functioning in professional or personal life rather than on ADHD symptoms. Thus detailed assessment becomes important in these cases to avoid misdiagnosis like depression, which may be overtly present as a comorbid condition. Despite the overall utility of stimulant and non-stimulant drugs in treating the majority of adults presenting with ADHD, there are limitations associated with drug treatment for this disorder. First, efficacy of drugs in these studies was documented as ‘effective’ for core symptoms, but there is little or almost no evidence that drug treatment has a positive effect on specific forms of functional impairment, such as poor motivation, disorganization, low self-esteem. Drug treatment alone may not be sufficient to remediate these deficits; some explicit skills and training in these areas may be necessary in adulthood. A significant subgroup of adults is apparently nonresponsive to drug treatment and thus also requires use of alternate interventions. Overall, the diagnosis and treatment of the disorder in adults can be a challenge because recent and integrative clinical guidelines are lacking. Keeping in mind that medication may not be fully effective for overall treatment of ADHD, especially of the associated functional impairment, researchers are looking towards other integrative therapeutic methods for treatment of adult ADHD. Neurocognitive psychotherapy is one of the models that have been developed, combining aspects of cognitive/behavioral therapy and cognitive rehabilitation that address both the neurocognitive and psychological aspects of ADHD. With this background, the following case study is reported to show the efficacy of neurocognitive psychotherapy in the treatment of adults with ADHD.

## CASE REPORT

A 28-year-old man, educated up to class XII and working as a sales representative in a private company, was referred by a private psychiatrist for assessment and further intervention with regard to complaints of difficulty in sustaining attention in any work, difficulty in concentrating during conversation, difficulty in organizing and planning, low self-esteem, worries about his performance in his job and poor motivation. The patient had been under medication without showing noticeable improvement. According to the patient, he did not have any problems during his childhood and adolescence. He was average in studies and did not like studies very much, so he left studies after passing class XII and joined a company as a sales representative at the age of 22.

### Tools

Following assessment, tools were administered to assess the patient’s symptoms and functioning level at different stages, viz., at the start of therapy, post-intervention and at follow-up.

### Conners’ adult attention-deficit hyperactivity disorder rating scale

The CAARS is a standardized tool designed to help in assessing, diagnosing and monitoring treatment of ADHD in adults.

### Trail-making test

The TMT is a test for sustained and divided attention, Originally constructed in 1938. It is composed of 2 parts, A and B. Part A consists of 25 circles printed on a sheet of paper. Each circle contains a number from 1 to 25. The subject’s task is to connect the circles with a pencil line as quickly as possible, beginning with the number 1 and proceeding in numerical sequence. Part B consists of 25 circles numbered from 1 to 13 and lettered from A to L. The task in Part B is to connect the circles in a sequence, alternating between numbers and letters. The scores represent the time required to complete each part.

### Stroop test

The Stroop neuropsychological screening test (SNST) is an efficient and sensitive neuropsychological screening measure based on the Stroop procedure. The test has been standardized and validated for adults (18 years and older). Although the test is a screening tool for brain damage, it efficiently assesses environmental dependency and poor response inhibition by the subjects.

### Wisconsin card-sorting test

The WCST was originally developed to assess abstract reasoning ability and the ability to shift cognitive strategy in response to changing environmental contingencies. WCST is considered a standard measure of “executive function,” requiring strategic planning, organized searching, utilizing environmental feedback to shift cognitive sets, directing behavior towards achieving a goal and modulating impulsive responding. It consists of 4 stimulus cards and 2 identical decks of 64 response cards with Figures of varying colors, forms and number. The subject’s task is to match the response cards to the stimulus cards based upon different criteria that keep on changing.

### Methods

#### The therapeutic package

The therapeutic program for this case is based on application of cognitive and behavioral principles and includes components intended to provide cognitive training in specific skills; reinforce and shape positive behavior; and impart new cognitions to maintain adaptive self-management behavior. The design of strategies was informed by neuropsychological theory and research concerning the core deficits in ADHD; thus, specific strategies were aimed to circumvent and compensate for deficits in sensitivity, delayed reinforce and in resistance to cognitive interference (Barkley, 1997; Nigg, 2005). The psychotherapy package was spanned over 10 weeks. Emphasis was put on practicing and maintaining of these strategies in daily life.

#### Psychoeducation

The patient was imparted psychoeducation to enable him to understand his symptoms as an outgrowth of a treatable disorder and not as his personality faults. In most cases, psychoeducation brings about great relief to patients and encourages them to pursue treatment. Emphasis was laid upon better understanding of the condition and learning to manage symptoms rather than fighting with them.

#### Cognitive training

To enhance attention, the following strategies were used:

- Letter cancellation task: The patient was demonstrated and made to practice letter cancellation task with increasing complexity. Homework practice was also assigned, even using newspapers. Improvement was assessed by gradually decreasing the time limit.
- Matching shapes in initial session (shapes becoming more complex in nature as session progressed).
- Making meaningful words from jumbled-up words.
- Abstracting articles into themes or major points after reading them from a newspaper or a book.

#### Cognitive-behavioral therapy

Considering patients’ occupational nature, organizational and planning skills were imparted emphasizing on the following aspects:

- Planning daily schedule for the present and the following day. At the end of the day, review of tasks completed was done.
- Breaking tasks into chunks and taking regular breaks: This helped in sustaining attention on a particular task, as well as eased the burden of doing it in a single spell.
- Maintaining a diary and ‘To-do’ list: Though the patient was using such technique earlier, it was regularized as daily activity.
- Prioritizing tasks and time management: Owing to variable workload, the patient was advised prioritizing the tasks and accordingly manage time.

#### Enhancing self-esteem and reducing negative thoughts

To improve the patient’s low self-esteem and poor motivation, he was helped in identifying his dysfunctional beliefs and their role in overall symptomatology. Emphasis was laid upon the following aspects:

- Identifying troubling conditions
- Becoming aware of beliefs and thoughts
- Pinpointing negative or inaccurate thoughts
- Challenging negative or inaccurate thoughts
- Changing thoughts and beliefs
- Using hopeful statements
- Focusing on the positive
- Re-labeling upsetting thoughts
- Encouraging himself

Table 1 shows overall improvement in all domains of CARSS across time following intervention and at follow-up. However, the score in inattention at follow-up was a little higher than that at post-intervention stage. In neurocognitive measures, the patient showed improvement in most of the domains after intervention. The scores though declined to some extent in some of the domains still were better than the baseline scores. Follow-up scores on Stroop test, categories completed and failure to maintain set in the WCST improved further at follow-up.

**Table 1:** Conners adult atttention-deficit hyperactivity disorder rating scales scores and neurocognitive measures of the patient at baseline, post-intervention and follow-up

Test	Measures	Baseline	Post-intervention	Follow-up
CAARS-SL	Inattention	78	52	61
	Impulsivity	64	59	55
	Hyperactivity	53	45	39
	Problem with self-concept	76	62	59
TMT	Part A	32 sec	15 sec	19 sec
	Part B	95 sec	62 sec	70 sec
STROOP	No. of correct responses	81	106	108
WCST	Trials administered	116	96	100
	Total no. of Correct responses	55	70	65
	Perseverative error	37	15	22
	Non-perseverative error	24	11	13
	Categories completed	3	4	4
	Failure to maintain set	3	2	2

## DISCUSSION

The patient had come with mixed set of problems. However, assessment revealed that inattention was the predominant problem that resulted in poor performance in job and decline in professional domain, further resulting in poor motivation and self-esteem. Findings in this case support the use of structured, combined approach of psychoeducation, Cognitive behaviour therapy (CBT) and cognitive training-based interventions; this approach was found to be effective in the patient and would be effective for adults with ADHD. Along with these elements, emphasis on regular practice and maintenance of these strategies in daily life is required to sustain the efficacy. Analyzing different components of intervention in this case, psychoeducation proved to be a vital part for the patient to get insight into the problem. Patient’s initial reliance on pharmacological intervention weakened by wider understanding of the problem and made space for inquiry about viable alternatives and made the patient to look upon the situation as treatable. The components of CBT mainly helped to improve the organizing skills of the patient in daily life; and to achieve overall improvement in self-esteem of the patient, motivation level and control over self-beliefs that helped maintain the symptoms. Cognitive training was the most vital part of the intervention package, which aimed upon improving attention, the principal area of intervention in ADHD. Findings show that the patient’s performance on neurocognitive measures declined to some extent at follow-up after initial improvement, suggesting continuous and prolonged practice to be a possible remedy for this. Finally the case supports neurocognitive psychotherapy, along with cognitive-behavioral and psychoeducative measures, as being effective in management of adult ADHD. It also indicated the need for long-term intervention program for sustained change in the patient’s behavior. These treatments, however, require further study for replication, extension and refinement.
